# Genomic Analysis of *Bacillus licheniformis* CBA7126 Isolated from a Human Fecal Sample

**DOI:** 10.3389/fphar.2017.00724

**Published:** 2017-10-13

**Authors:** Changsu Lee, Joon Yong Kim, Hye Seon Song, Yeon Bee Kim, Yoon-E Choi, Changmann Yoon, Young-Do Nam, Seong Woon Roh

**Affiliations:** ^1^Microbiology and Functionality Research Group, World Institute of Kimchi, Gwangju, South Korea; ^2^Division of Environmental Science & Ecological Engineering, Korea University, Seoul, South Korea; ^3^Research Group of Gut Microbiome, Korea Food Research Institute, Sungnam, South Korea; ^4^Department of Food Biotechnology, University of Science and Technology, Daejeon, South Korea

**Keywords:** *Bacillus licheniformis*, genome sequence, human fecal sample, stress response genes, multilocus sequence typing

## Introduction

*Bacillus licheniformis* is a Gram-positive, endospore-forming, saprophytic organism that occurs in plant and soil (Veith et al., 2004). A taxonomical approach shows that it is closely related to *Bacillus subtilis* (Lapidus et al., 2002; Xu and Côte, 2003; Rey et al., 2004). Generally, most bacilli are predominantly aerobic; however, *B. licheniformis* is a facultative anaerobe compared to other bacilli in ecological niches (Alexander, 1977). The commercial utility of the extracellular products of *B. licheniformis* makes this microorganism an economically interesting species (Kovács et al., 2009). For example, *B. licheniformis* is used industrially for manufacturing biochemicals, enzymes, antibiotics, and aminopeptidase. Several proteases such as α-amylase, penicillinase, pentosanase, cycloglucosyltransferase, β-mannanase, and certain pectinolytic enzymes are synthesized industrially using *B. licheniformis* (Rodríguez-Absi and Prescott, 1978; Rey et al., 2004). The proteases are used in the detergent industry and the amylases are utilized for starch hydrolysis, desizing of textiles, and sizing of paper (Erickson, 1976). In addition, certain strains are utilized to produce peptide antibiotics, specialty chemicals, and poly-γ-glutamic acid (Nierman and Maglott, 1989; Rey et al., 2004).

The annotated genome sequence of *B. licheniformis* has been previously analyzed to assess the biotechnological importance of the organism (Veith et al., 2004). Since the first sequencing, the genomes of specific *B. licheniformis* strains have been sequenced to completely realize its industrial potential. In this study, genome sequencing of *B. licheniformis* CBA7126 isolated from a human fecal sample was performed to understand bacterial specificity. The genome sequence of CBA7126 revealed features such as stress response genes, antibiotic-resistance genes, and genes for resistance to toxic compounds, which are of considerable biotechnological value.

## Materials and methods

### Bacterial isolation, culture conditions, and DNA extraction

*B. licheniformis* CBA7126 was isolated from the feces of a 74-year-old man in Geochang-gun, South Korea and was cultured under anaerobic conditions in Gifu Anaerobic Medium (GAM) (containing per liter of deionized distilled water: 10 g peptone, 3 g soytone, 10 g proteose peptone, 13.5 g bovine serum albumin, 5 g yeast extract, 2.2 g beef extract, 2.5 g monopotassium phosphate, 1.2 g liver extract, 3 g sodium chloride, 0.3 g l-cystein, 0.3 g sodium thioglychollate, 3 g dextrose, 5 g soluble starch) at 37°C for 48 h. Genomic DNA of strain CBA7126 was extracted using the QIAamp DNA extraction kit (Qiagen, USA) and QuickGene DNA tissue kit S (Kurabo, Japan), and purified using the MG genomic DNA purification kit (Doctor Protein, Korea) according to the manufacturer's instructions. The purity and concentration of the extracted genomic DNA were measured using the Nanodrop spectrophotometer (NanoDrop Technologies, UK).

### Genome sequencing, assembly, and annotation

The genome of *B. licheniformis* CBA7126 was sequenced using a 20-kb SMRTbell library and PacBio RS II system (Pacific Biosciences, USA), and *de novo* assembly was performed using the HGAP2 protocol in PacBio SMRT Analysis version 2.3.0. rRNAs and tRNAs were analyzed using RNAmmer 1.2 (Lagesen et al., 2007) and tRNAscan-SE 1.21 (Lowe and Eddy, 1997), respectively. The potential coding regions and functional genes were predicted via a combination of Glimmer 3.02 (Delcher et al., 1999), COG database (Tatusov et al., 2003), the Rapid Annotation Search Tool (RAST) (Aziz et al., 2008), and the National Center for Biotechnology Information (NCBI) prokaryotic genome annotation pipeline (PGAP) 4.1 (Tatusova et al., 2016). Prophages in the genome were identified using the PHAge Search Tool (PHAST) (Zhou et al., 2011). In addition, pathogenicity of strain CBA7126 was predicted using PathogenFinder 1.1 (Cosentino et al., 2013). Carbohydrate-active enzymes were annotated using dbCAN (Yin et al., 2012).

### Comparative genomic analysis

To identify the unique features of strain CBA7126, the genomes of *B. licheniformis* and *Bacillus* sp. strains (*B. licheniformis* B4164, *B. licheniformis* VTM3R78, *B. licheniformis* V30, *B. licheniformis* B4124, and *Bacillus* sp. H15-1) were selected for comparative genomic analysis using the NCBI genome database (http://www.ncbi.nlm.nih.gov/genome/). For calculation of overall genome relatedness, average nucleotide identity (ANI), and orthologous average nucleotide identity (OrthoANI) analysis of *B. licheniformis* CBA7126 was performed on sequences of related species using the ANI calculator (http://enve-omics.ce.gatech.edu/ani/) and orthologous average nucleotide identity tool (OAT) of ChunLab (Lee et al., 2016). The genome structure of strain CBA7126 was compared to those of *B. licheniformis* B4164 (LQYQ00000000.1), *B. licheniformis* VTM3R78 (FOFE00000000.1), *B. licheniformis* V30 (LQRR00000000.1), *Bacillus* sp. H15-1 (CP018249.1), and *B. licheniformis* B4124 (LKPQ00000000.1) having symmetric identity >97% with strain CBA7126, using the alignment program MAUVE (Darling et al., 2004). Pan-genome Orthologous Groups (POGs) were analyzed using BIOiPLUG Comparative Genomics Database (https://www.bioiplug.com/). Venn diagram was constructed based on the number of POGs of strain CBA7126 and the related strains. Clustered regularly interspaced short palindromic repeats (CRISPR) was analyzed using CRISPRfinder (Grissa et al., 2007).

### Multilocus sequence typing (MLST)

Multilocus sequence typing (MLST) analysis based on internal sequences of *adk, ccpA, recF, rpoB, spo0A*, and *sucC* genes was performed (Larsen et al., 2012; Madslien et al., 2012). The MLST sequence type of strain CBA7126 was determined using the MLST 1.8 database (https://cge.cbs.dtu.dk/services/MLST/) of *B. licheniformis* (Larsen et al., 2012).

### Ethics approval

The study protocol was approved by the institutional review board of the Theragen ETEX Bio Institute (700062-20160804-JR-005-02).

## Results

### General genomic features of *B. licheniformis* CBA7126

**Figure 1:**
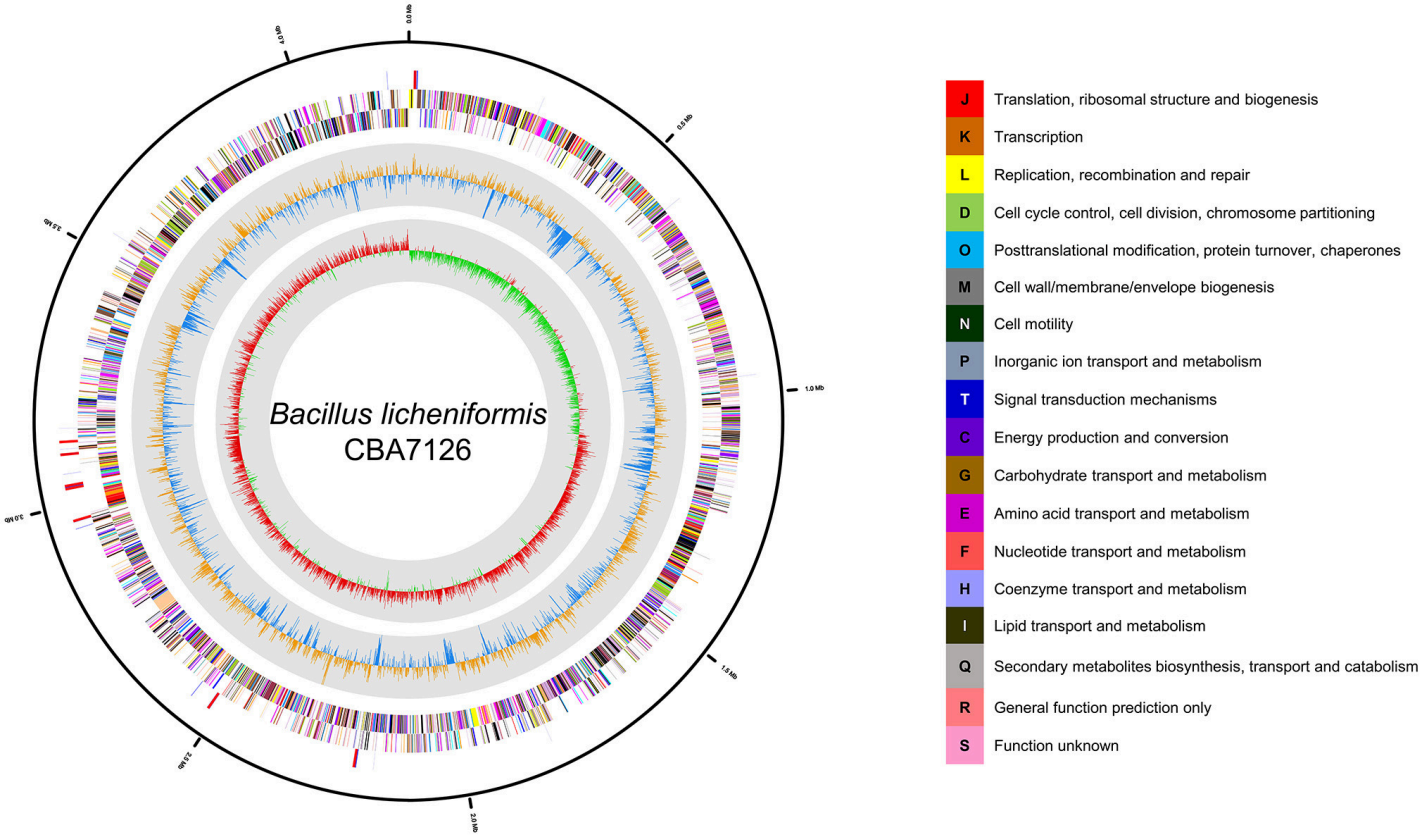
Graphic circular map of the *Bacillus licheniformis* CBA7126 genome. The outer circle shows RNA genes (red, tRNA; blue, rRNA) and genes on the sense and antisense strands (colored according to COG categories), shown from the outside of the circle to the center. The inner circle shows the GC skew, with yellow and blue indicating positive and negative values, respectively; the GC content is indicated in red and green. This genome map was visualized using CLgenomics 1.55 (Chun Lab Inc.).

The genome of *B. licheniformis* CBA7126 was 4,216,391 bp long with a G + C content of 46.24 mol% (Table 1). The genome is predicted to contain two contigs of 4,209,959 and 6,972 bp. Strain CBA7126 genome contained 4,276 coding sequences, 24 rRNA genes (8 of the 16S-5S-23S RNA gene operon), and 81 tRNA genes (Figure 1). For functional classification, the genome of strain CBA7126 was analyzed using the Cluster of Orthologous Groups (COG) database (http://www.ncbi.nlm.nih.gov/COG/), and 3,743 genes were annotated. The annotated genes belonged to the following categories: function unknown (S; 884 genes), general function prediction only (R; 344), transcription (K; 319 genes), carbohydrate transport, and metabolism (G; 316 genes), amino acid transport and metabolism (E; 298 genes), inorganic ion transport and metabolism (P; 219 genes), energy production and conversion (C; 180 genes), replication, recombination, and repair (L; 140 genes), and secondary metabolite biosynthesis, transport, and catabolism (Q; 62 genes) (Supplementary Table 1). In addition, SEED viewer version 2.0 revealed that >9% of the major categories contained genes required for metabolism of “carbohydrates” (610 genes), “amino acids and derivatives” (457 genes), and “cofactors, vitamins, prosthetic group, pigments” (280 genes) (Supplementary Figure 1). A total of 193 CAZyme-encoding genes were annotated using dbCAN, including five for auxiliary activities (AAs), 39 for carbohydrate-binding modules (CBMs), 36 for carbohydrate esterases (CEs), 68 for glycoside hydrolases (GHs), 39 for glycosyl transferases (GTs), and 6 for polysaccharide lyases (PLs).

**Table 1:** Features of the *Bacilus licheniformis* CBA7126 genome.

	***B. licheniformis* CBA7126**
Sequencing platform	PacBio RS II system
Assembler	PacBio SMRT Analysis 2.3.0
Assembly accession	GCA_001950175.1
Methods reads	90,824
Assembly size (bp)	4,216,931
Contig numbers	2
N50	4,209,959
L50	1
Genome coverage	319.23
DNA G + C content (mol%)	46.24
CDSs	4,276
rRNA number	24
tRNA number	81
Genes assigned to COGs	3,743
CRISPRs	0

### Comparative genomic data

Analysis of the orthoANI values among *Bacillus* genome sequences with symmetric identity of >97% revealed that *B. licheniformis* CBA7126 has higher than 99% genome sequence similarity with other species. The genome of strain CBA7126 was closest to that of *B. licheniformis* VTM3R78 (99.99% orthoANI), followed by *B. licheniformis* B4164 (99.98%), *Bacillus* sp. H15-1 (99.85%), *B. licheniformis* B4124 (99.81%), and *B. licheniformis* V30 (99.80%) (Supplementary Figure 2). Similar results were also obtained using ANI. Based on the results of Lee et al. (2016), similarity values >95–96% indicated that two strains belong to the same species. Therefore, strain CBA7126 was confirmed to be a species of *B. licheniformis*. The genome of strain CBA7126 was aligned with more than 97% symmetric identity with those of strains *B. licheniformis* B4164, *B. licheniformis* VTM3R78, *B. licheniformis* V30, *Bacillus* sp. H15-1, and *B. licheniformis* B4124 using MAUVE. The genomic representations of the other strains were rearranged based on the structure of strain CBA7126. Gene order comparison was established for seven regions with Local Collinear Blocks (LCBs). The structure of strain CBA7126 was similar to that of *B. licheniformis* B4124 and *B. licheniformis* V30 (Supplementary Figure 3). Comparison of strain CBA7126 genomic structure with that of *Bacillus* sp. H15-1 showed that two major regions were in opposite direction. Analysis based on the POG of strain CBA7126 and the closely related strains identified 4,108 shared genes and 137 unique genes (Supplementary Figure 4). Strain CBA7126 possessed 19 genes among the unique genes: 1 poly (glycerol-phosphate) alpha-glucosyltransferase, 1 thymidylate synthase (FAD), 2 prophage-derived protein, and 15 hypothetical proteins. The three genes among the 19 unique genes of strain CBA7126 were classified to one carbon pool by folate, pyrimidine metabolism, and metabolic pathways, based on KEGG analysis. In addition, CRISPR analysis indicated that strain CBA7126 did not harbor any known CRISPRs.

### Phage and pathogenesis-related genes

PHAST analysis was performed for identifying prophage contamination in the genome sequence of strain CBA7126. Contig 1 contained three intact and two incomplete prophages, whereas contig 2 contained only one incomplete prophage (Supplementary Figure 5). Intact regions of prophages were located between positions 1,596,547–1,623,555, 1,775,723–1,820,161, and 3,429,284–3,483,201 bp, respectively. Strain CBA7126 was identified to be a human pathogen with 0.81 probability in PathogenFinder 1.1. Analysis of pathogenesis-related genes showed that all the 238 analyzed genes encoded pathogenesis-associated proteins.

### Multilocus sequence typing (MLST) analysis

MLST analysis of strain CBA7126 was performed using six housekeeping genes (*adk, ccpA, recF, rpoB, spo0A*, and *sucC*). MLST analysis showed that strain CBA7126 belonged to sequence type 3 since this organism harbored *adk_2, ccpA_1, recF_1, rpoB_1, spo0A_1*, and *sucC_2* (Supplementary Table 2). Previously reported isolates of sequence type 3 are *B. licheniformis* NVH1023, F5520, CCUG41412, NVH1111, NVH1113, LMG17661, and M3.

### Stress response genes and resistance to toxic compounds

Comparison with NCBI PGAP 4.1 showed that the genome of strain CBA7126 harbors several stress response genes and various genes required for resistance to antibiotics and toxic compounds (Tatusova et al., 2016). The identified stress tolerance genes encode general stress proteins (WP_003179040.1; WP_009329495.1; WP_011198337.1; WP_003186243.1), universal stress proteins (WP_011197701.1; WP_003178013.1), cold shock proteins (WP_003153604.1; WP_003179166.1), and the UV-damage repair protein UvrX (WP_003183238.1). These genes were closely associated with the survival of bacteria in the natural environment. The genes identified for resistance to toxic compounds encode monooxygenase (required for antibiotic resistance) (WP_017473926.1; WP_003181975.1), l-asparaginase (WP_003183042.1; WP_003183042.1; WP_061565867.1), the multidrug resistance protein NorM (WP_009328059.1), YkkD (WP_003180981.1), YkkC (WP_003180979.1), arginase (WP_009330115.1; WP_009330115.1; WP_003178878.1; WP_003178436.1), chemical damaging agent resistance protein C (WP_017474008.1; WP_003178723.1), toxic anion resistance protein (WP_003178733.1), lantibiotic-related proteins (WP_003186355.1; WP_003186351.1; WP_003186379.1; WP_003186381.1), bacitracin, and various proteins of the ABC transporter family. Among the genes related to stress response, l-asparaginase, arginase, lantibiotic, and bacitracin are used for industrial application.

## Data access

The genome sequence of *B. licheniformis* CBA7126 has been deposited in DDBJ/ENA/GenBank under the accession numbers BDJJ01000001–BDJJ01000002.

## Author contributions

SR and YN designed and coordinated all the experiments. HS performed cultivation, DNA extraction, and purification. CL, JK, HS, YK, YC, and CY performed the sequencing, genome assembly, gene prediction, gene annotation, and comparative genomic analysis. CL, YN, and SR wrote manuscript. All authors have read and approved the manuscript.

### Conflict of interest statement

The authors declare that the research was conducted in the absence of any commercial or financial relationships that could be construed as a potential conflict of interest.
