# Supplementary material for: Genomic Analysis of Bacillus licheniformis CBA7126 Isolated from a Human Fecal Sample
Source: Front Pharmacol. 2017 Oct 13;8:724. doi: 10.3389/fphar.2017.00724 (PMC5645497; doi:10.3389/fphar.2017.00724)
Supplement: Supplementary file 1 [file DataSheet1.DOCX]

Supplementary Material

**Genomic Analysis of *Bacillus licheniformis* CBA7126 Isolated from a Human Fecal Sample**

**Changsu Lee^1^, Joon Yong Kim^1^, Hye Seon Song^1^, Yeon Bee Kim^1^, Yoon-E Choi^2^, Changmann Yoon^2^, Young-Do Nam^3,4*^ and Seong Woon Roh^1*^**

*** Correspondence:**

Seong Woon Roh

seong18@gmail.com;

Young-Do Nam

youngdo98@kfri.re.kr

# Supplementary Figures and Tables

## Supplementary Figures

**Supplementary Figure S1.** The subsystem category distribution of *Bacillus licheniformis* CBA7126. The chart represents the coverage of proteins which were grouped into subsystems. A total of 3,086 proteins were categorized within these subsystems.

**
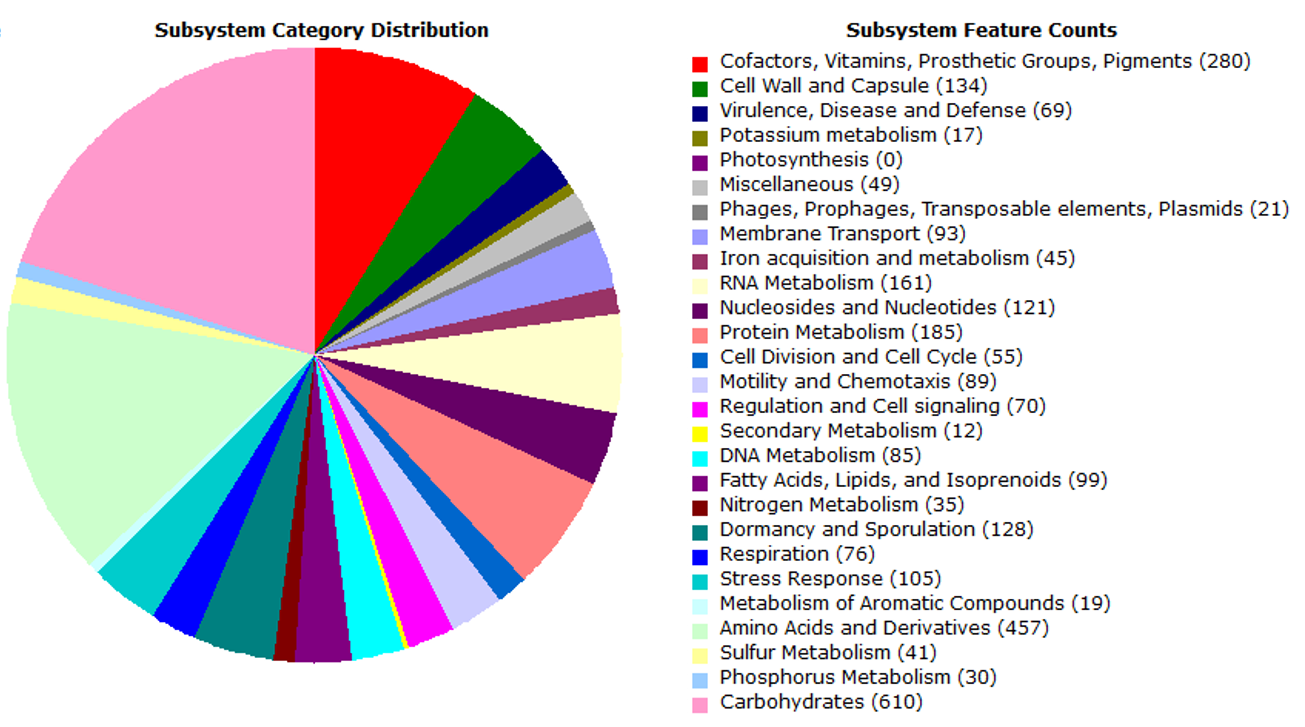
**

**Supplementary Figure S2.** The orthoANI results calculated using species of symmetric identity over 97%: *Bacillus licheniformis* CBA7126, *B. licheniformis* VTM3R78, *B. licheniformis* B4164, *Bacillus* sp. H15-1, *B. licheniformis* B4124, and *B. licheniformis* V30.

**
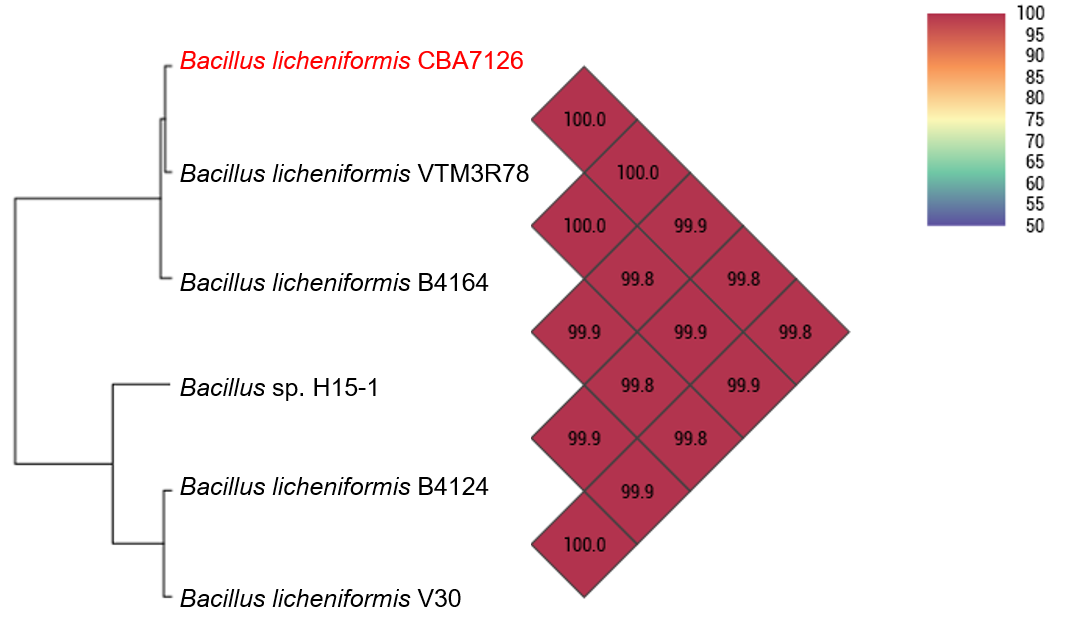
**

**Supplementary Figure S3.** Multiple alignment of strain CBA7126, VTM3R78, B4164, B4124, V30 and *Bacillus* sp. H15-1 genomes. Schematic of the 6 genomes were visualized Local Colinear Blocks (LCBs) of the sequences. Based on strain CBA7126 structure, the genomic representations of other strains were rearranged. LCBs are represented by blocks of different colors.

**
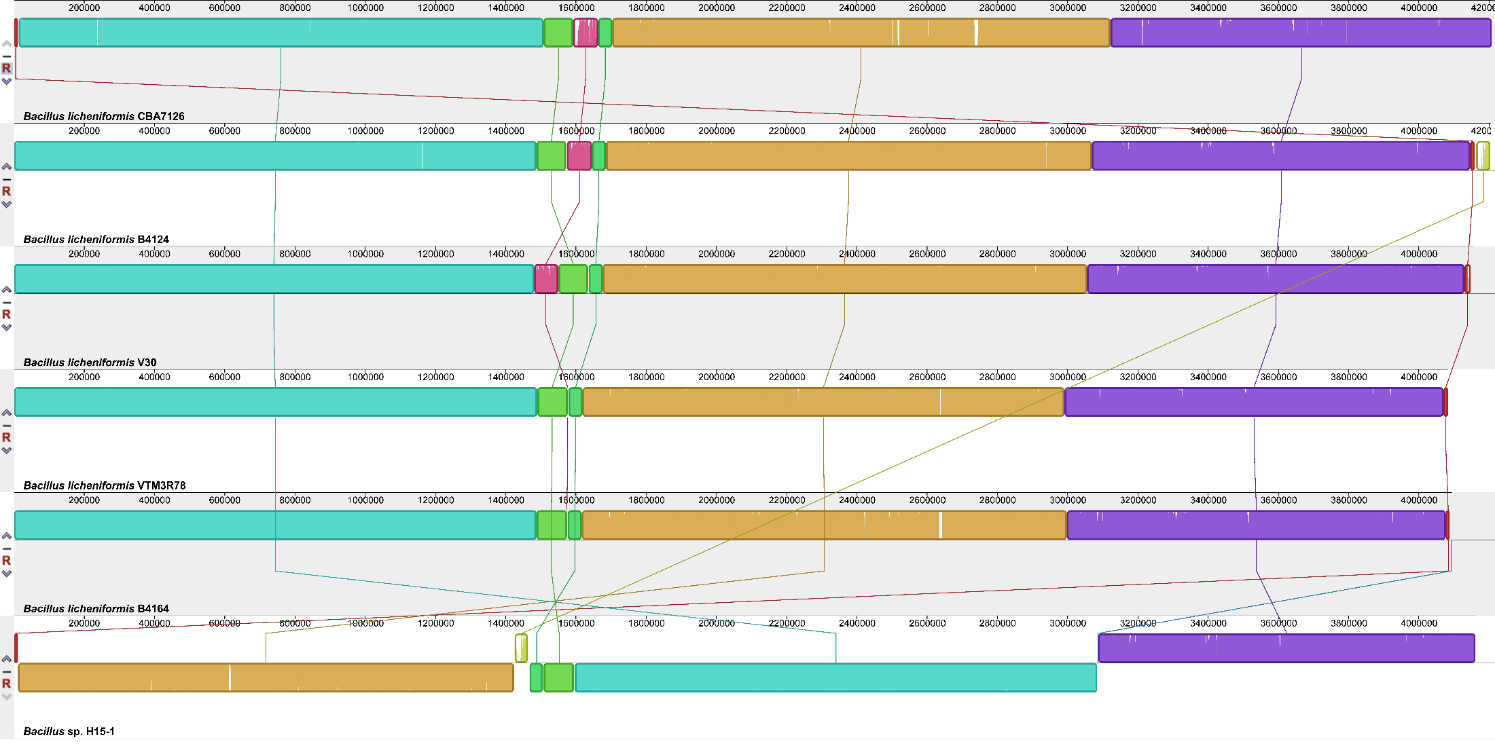
**

**Supplementary Figure S4.**Venn diagram representing the number of shared and unique genes based on POGs of *Bacillus licheniformis* CBA7126, VTM3R78, B4164, B4124, V30, and *Bacillus* sp. H15-1.


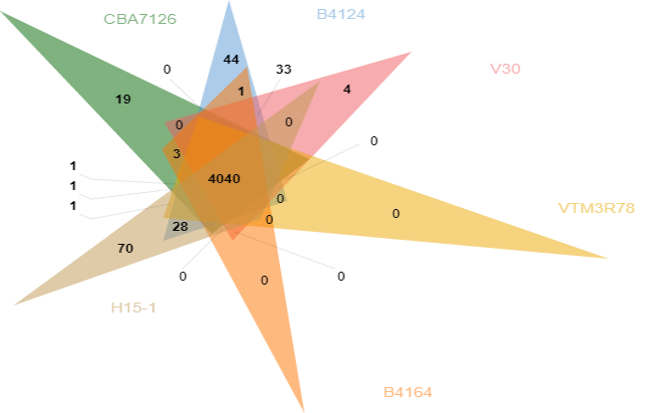


**Supplementary Figure S5.** Prophages of *Bacillus licheniformis* CBA7126 were identified using the PHAse Search Tool (PHAST). (A) Contig 1 of strain CBA7126 contained 3 intact prophages (red color) and 2 incomplete prophages (gray color). (B) Contig 2 of strain CBA7126 contained only 1 incomplete prophage (gray color).


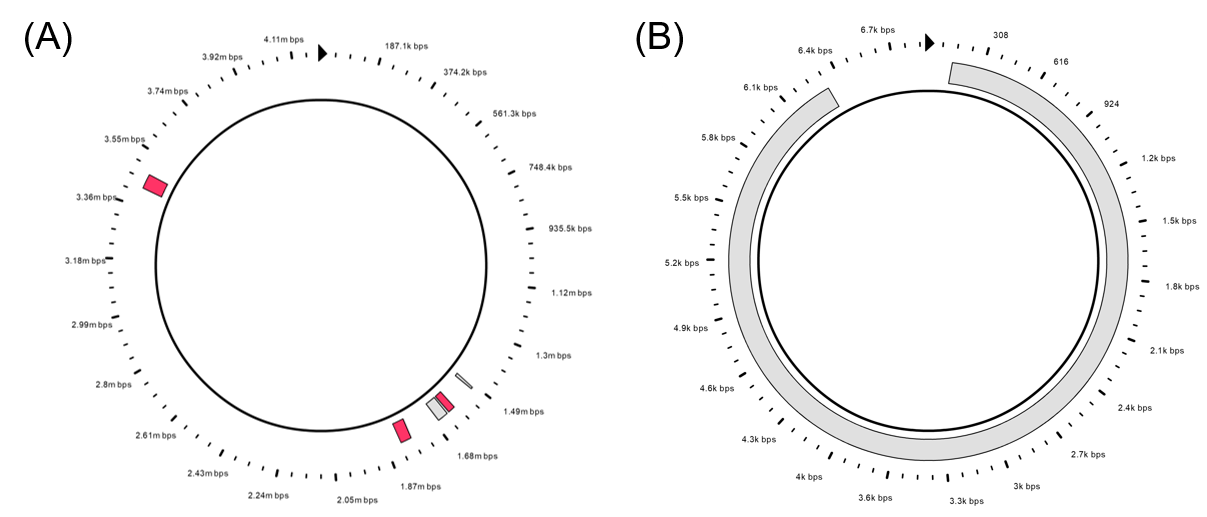


## Supplementary Tables

**Supplementary Table S1.** Genes associated with general COG functional categories in the genome of *Bacillus licheniformis* CBA7126.

| COG | Description | Number of genes | % |
| --- | --- | --- | --- |
| J | Translation, ribosomal structure, and biogenesis | 162 | 4.33% |
| K | Transcription | 319 | 8.52% |
| L | Replication, recombination, and repair | 140 | 3.74% |
| D | Cell cycle control, cell division, chromosome partitioning | 35 | 0.94% |
| O | Post-translational modification, protein turnover, chaperones | 108 | 2.89% |
| M | Cell wall/membrane/envelope biogenesis | 183 | 4.89% |
| N | Cell motility | 52 | 1.39% |
| P | Inorganic ion transport and metabolism | 219 | 5.85% |
| T | Signal transduction mechanisms | 150 | 4.01% |
| C | Energy production and conversion | 180 | 4.81% |
| G | Carbohydrate transport and metabolism | 316 | 8.44% |
| E | Amino acid transport and metabolism | 298 | 7.96% |
| F | Nucleotide transport and metabolism | 87 | 2.32% |
| H | Coenzyme transport and metabolism | 103 | 2.75% |
| I | Lipid transport and metabolism | 101 | 2.70% |
| Q | Secondary metabolites biosynthesis, transport and catabolism | 62 | 1.66% |
| R | General function prediction only | 344 | 9.19% |
| S | Function unknown | 884 | 23.62% |
| Total |  | 3743 | 100% |

**Supplementary Table S2.** Genotypes representing results of *Bacillus licheniformis* CBA7126 using multilocus sequence typing (MLST).

| \| Locus \| % Identity \| HSP Length \| Allele Length \| Gaps \| Allele \| \| --- \| --- \| --- \| --- \| --- \| --- \| \| *adk* \| 100.00 \| 465 \| 465 \| 0 \| *adk_2* \| \| *ccpa* \| 100.00 \| 561 \| 561 \| 0 \| *ccpA_1* \| \| *recf* \| 100.00 \| 561 \| 561 \| 0 \| *recF_1* \| \| *rpob* \| 100.00 \| 495 \| 495 \| 0 \| *rpoB_1* \| \| *spo0a* \| 100.00 \| 558 \| 558 \| 0 \| *spo0A_1* \| \| *succ* \| 100.00 \| 549 \| 549 \| 0 \| *sucC_2* \| |
| --- | --- | --- | --- | --- | --- | --- | --- | --- | --- | --- | --- | --- | --- | --- | --- | --- | --- | --- | --- | --- | --- | --- | --- | --- | --- | --- | --- | --- | --- | --- | --- | --- | --- | --- | --- | --- | --- | --- | --- | --- | --- | --- |
